# Interrelationships of disease activity, central sensitization, psychosocial and lifestyle factors in axial spondyloarthritis

**DOI:** 10.1093/rheumatology/keaf102

**Published:** 2025-02-22

**Authors:** Stan C Kieskamp, Yvonne van der Kraan, Suzanne Arends, Fréke Wink, Reinhard Bos, Roy Stewart, Davy Paap, Anneke Spoorenberg

**Affiliations:** Department of Rheumatology and Clinical Immunology, University Medical Centre Groningen, Groningen, The Netherlands; Department of Rheumatology and Clinical Immunology, University Medical Centre Groningen, Groningen, The Netherlands; Department of Rheumatology and Clinical Immunology, University Medical Centre Groningen, Groningen, The Netherlands; Department of Rheumatology, Medical Centre Leeuwarden, Leeuwarden, The Netherlands; Department of Rheumatology, Medical Centre Leeuwarden, Leeuwarden, The Netherlands; Department of Health Sciences, Community and Occupational Medicine, University Medical Centre Groningen, Groningen, The Netherlands; Department of Rheumatology and Clinical Immunology, University Medical Centre Groningen, Groningen, The Netherlands; Department of Physiotherapy, Saxion University of Applied Sciences, Enschede, The Netherlands; Department of Rheumatology and Clinical Immunology, University Medical Centre Groningen, Groningen, The Netherlands

**Keywords:** axial spondyloarthritis, disease activity, central sensitization, biopsychosocial, lifestyle, illness perception

## Abstract

**Objectives:**

In a substantial portion of patients with axial SpA (axSpA), disease activity scores remain high despite anti-inflammatory treatment. This is possibly due to factors beyond active inflammation including different pain mechanisms and psychosocial factors. Therefore, our aim was to build a biopsychosocial model to explore the interrelationships of Axial Spondyloarthritis Disease Activity Score (ASDAS) with central sensitization, psychological and lifestyle factors in patients with axSpA.

**Methods:**

Consecutive patients from the prospective Groningen Leeuwarden axSpA (GLAS) cohort were included in this cross-sectional study. Assessments included in the model were educational level, BMI, questionnaires on central sensitization, illness perception, pain catastrophizing, coping, anxiety and depression, physical activity (modified Short QUestionnaire to ASsess Health-enhancing physical activity, mSQUASH) and ASDAS. Structural equation modelling (SEM), a multivariate analysis testing hypothesized interrelationships between variables, was applied to investigate the effects of central sensitization, psychosocial and lifestyle factors on ASDAS.

**Results:**

A total of 332 consecutive axSpA patients were eligible for analyses, of whom 59% were male; median symptom duration was 21 years and mean ASDAS was 2.2 ± 0.9. The final SEM model had a satisfactory fit [root mean square error of approximation = 0.057 (95% CI 0.45–0.70), comparative fit index = 0.936]. Illness perception, central sensitization and BMI had direct, significant, effects on ASDAS. Psychological well-being and educational level were significantly indirectly associated with ASDAS through illness perception.

**Conclusion:**

Our analyses exploring the interrelationships of biopsychosocial factors related to ASDAS showed that factors beyond inflammation, especially illness perception and central sensitization, seem to contribute significantly to ASDAS in patients treated for axSpA in our standard-of-care cohort, confirming the need for a biopsychosocial approach.

Rheumatology key messages

- In a standard-of-care axSpA patients cohort, ASDAS reflects more than inflammation alone.
- Psychosocial factors, illness perception and central sensitization affect ASDAS most in treated axSpA patients.
- A biopsychosocial approach is warranted in case of high disease activity on ASDAS despite treatment.

## Introduction

Axial SpA (axSpA) is a chronic inflammatory disorder affecting especially the sacroiliac joints and the spine. The disease starts at a relatively young age, causing symptoms such as chronic (inflammatory) back pain, fatigue and stiffness. The burden of axSpA is high and related to disease activity (DA), physical function, and health-related quality of life [1, 2].

The Axial Spondyloarthritis Disease Activity Score (ASDAS) [3] is the preferred and most often used assessment of DA in research and in daily clinical practice [4]. It combines patient-reported items with the general inflammation marker CRP. However, CRP is not elevated in all axSpA patients with disease-related inflammation, and in patients treated with anti-inflammatory medication, CRP is often low, whereas symptoms such as chronic pain can still be present. Unfortunately, a sensitive and specific biomarker reflecting inflammation in axSpA is still lacking. The patient-reported questions in ASDAS inquire about pain, fatigue and stiffness, are not specific for inflammation and may reflect also overall disease burden [5]. This aligns with the biopsychosocial model of health, where DA is seen as a multidimensional and dynamic interplay among biological, psychological and social factors [6].

In light of this biopsychosocial perspective, it is important to interpret pain in axSpA not only from a nociceptive origin, as non-nociceptive pain mechanisms may also play a role. There has been increasing focus in studies on neuropathic pain and nociplastic pain due to central sensitization. Central sensitization refers to the amplified response of the CNS to nociceptive or non-nociceptive signals, leading to sensory hypersensitivity including increased pain perception [7]. Recent studies have shown that 45–60% of patients with axSpA have a high probability of central sensitization [8]. Also, recent studies in axSpA using Quantitative Sensory Testing have confirmed the presence of altered somatosensory function related to central sensitization [9–11]. There is growing evidence suggesting that central sensitization needs to be considered when interpreting DA in axSpA, as central sensitization has been demonstrated to be independently associated with ASDAS [8].

Psychological factors such as illness perceptions, pain-related worrying, coping strategies and psychological well-being are key aspects within the biopsychosocial model of health in rheumatic diseases and may be associated with DA assessment. In RA, a direct association was found between illness perceptions, and pain and fatigue levels [12]. In addition, an exercise program in patients with chronic spinal pain aiming at changing illness perceptions resulted in a relevant improvement in pain severity when compared with normal exercise therapy [13]. In axSpA, negative illness perceptions have shown to impact disease activity (ASDAS) [8]. Negative illness perceptions and pain-related worrying may lead to passive (evasive) coping strategies to manage stressors such as pain, stiffness and fatigue [14], all of which are assessed by the ASDAS.

Lifestyle factors may also influence ASDAS. It is widely acknowledged that physical activity improves clinical DA assessments in rheumatic diseases [15]. Promoting physical activity is part of the Assessment of SpondyloArthritis international Society (ASAS) and EULAR recommendations for the management of axSpA [4]. In the general population, exercise has been shown to have an anti-inflammatory effect [16]. Conversely, a lack of physical activity can lead to obesity, which is prevalent among patients with axSpA [17]. Adipose tissue may contribute to an inflammatory environment through adipokine secretion. Furthermore, obesity is also associated with increased biomechanical strain, mood disturbance and poor sleep [18–20].

In summary, available research supports a significant impact of central sensitization, psychological factors and lifestyle factors on DA assessment in patients with axSpA. To better understand and interpret the multifaceted nature of assessing DA, and therefore ASDAS, a broad holistic biopsychosocial approach is needed. Therefore, our study aim was to build a biopsychosocial model of the interrelationships of ASDAS with central sensitization, illness perceptions, coping strategies, psychological well-being and lifestyle factors in patients with axSpA, using structural equation modelling (SEM).

## Methods

### Patients

For this cross-sectional analysis, consecutive outpatients from the prospective observational Groningen Leeuwarden Axial Spondyloarthritis (GLAS) cohort visiting the outpatient clinic of the Medical Centre Leeuwarden (secondary referral centre) and University Medical Centre Groningen (tertiary referral centre) were included. All axSpA patients in this cohort are treated according to the combined EULAR and ASAS guidelines. A detailed description of the GLAS cohort and assessments has been published previously [21].

### Assessments

Demographic and clinical assessments collected within the GLAS cohort were used for this study, including highest attained educational level, BMI and ASDAS.

The following additional questionnaires were used alongside these standardized assessments.

The Central Sensitization Inventory (CSI) [22, 23] assesses the likelihood of central sensitization being present and was validated in patients with axSpA. It consists of 25 items on a 5-point Likert scale about the presence of symptoms associated with central sensitization, with a total sum score ranging from 0 to 100. Higher scores indicate increased symptom frequency and are associated with an increased risk of central sensitization.

The Revised Illness Perception Questionnaire (IPQ-R) [24, 25] assesses the five components of illness representations in Leventhal’s self-regulatory model. The first part consists of 14 items, where the patient is asked whether they experience any of a list of 14 symptoms as a result of axSpA, leading to the ‘identity’ domain score ranging from 0 to 14. The second part of the IPQ-R consists of 38 items on a 5-point Likert scale divided into seven domains: timeline chronic (perceived chronicity of the disease; 6–30), consequences (perceived impact of the disease; 6–30), personal control (perceived personal control over the disease; 6–30), treatment control (perceived efficacy of treatment; 5–25), illness coherence (perceived understanding of their disease; 5–25), timeline cyclical (perceived variability of the disease; 4–20) and emotional representations (negative emotions due to the disease; 6–30). High scores on identity, timeline chronic, consequences, timeline cyclical and emotional representations can be considered detrimental, while high scores on personal/treatment control and illness coherence are beneficial.

The Pain Catastrophizing Scale (PCS) [26, 27] assesses the presence of pain-related worrying (catastrophizing thoughts concerning pain). It consists of 13 items on a 5-point Likert scale with a total sum score ranging from 0 to 52. Higher scores indicate more pain-related worrying.

The Coping with Rheumatic Stressors questionnaire (CORS) [28] measures the extent towards which certain coping styles are applied, and was developed in patients with RA. It consists of 61 items on a 4-point Likert scale about eight separate coping strategies, each pertaining to one of three chronic rheumatic stressors: three coping strategies for pain, three for limitations and two for dependency. Respectively, these coping strategies are comforting cognitions, decreasing activities, diverting attention, pacing, optimism, creative solution seeking, accepting one’s dependence and showing consideration. Sum scores are calculated for each of these eight coping strategies. High scores for a coping strategy indicate frequent utilization of this strategy.

The Hospital Anxiety and Depression Scale (HADS) [29, 30] detects states of depression and anxiety without considering somatic symptoms. The anxiety and depression subscales are also valid measures of severity of the emotional disorder. It consists of 14 items on a 4-point Likert scale about symptoms of anxiety and depression, with scores for each ranging from 0 to 21. Cutoff points were defined as 0–7 indicating no depression or anxiety, 8–10 indicating possible depression or anxiety, and 11–21 indicating probable depression or anxiety.

The modified Short QUestionnaire to ASsess Health-enhancing physical activity (mSQUASH) [31] is a questionnaire that assesses the amount and type of daily (aerobic) physical activity in four domains (commuting activities; work or school/study; household activities; leisure activities, sports and exercise) and was developed in patients with axSpA. Higher scores indicate a higher level of physical activity.

### Hypothesis and statistical analysis

**Figure 1.**
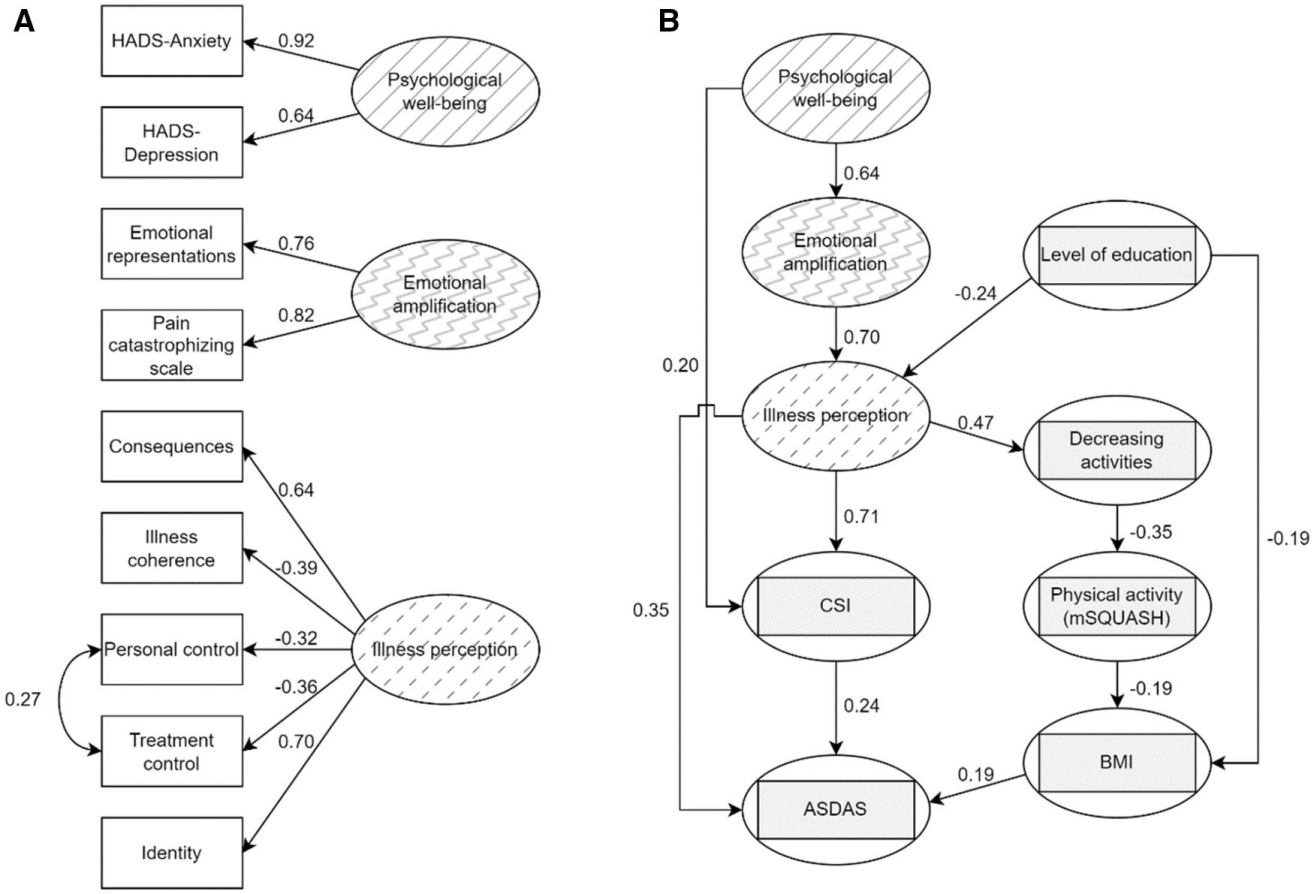
Graphical representation of structural equation model. Values shown are significant standardized parameter estimates. Ellipses contain latent variables; rectangles contain indicator variables; circumscribed rectangles contain observable variables. (A) Measurement model, (B) structural model. ASDAS: Ankylosing Spondylitis Disease Activity Score; CORS: Coping with Rheumatic Stressors questionnaire; CSI: Central Sensitization Inventory; HADS: Hospital Anxiety and Depresion Scale; mSQUASH: modified Short QUestionnaire to Assess Health-enhancing physical activity

After reviewing the literature to determine theorized relationships between the variables of interest in relation to ASDAS, we formulated our hypothesis by drawing up a theoretical model based on a biopsychosocial framework to be tested with SEM (deductive approach) (Supplementary Fig. S1, available at *Rheumatology* online). SEM consists of two main parts: the measurement model and the structural model. The measurement model defines latent variables, which are variables that are not directly observable or measurable but are inferred from other observable variables (also known as indicators, such as questionnaires items). These latent variables represent underlying constructs or concepts that cannot be directly measured. In our model, the latent variables are psychological well-being, emotional amplification and illness perception (see Fig. 1A). In the structural model the latent variables are combined with observable variables, which in our model are level of education, CSI, coping, mSQUASH, BMI and ASDAS (Fig. 1B). The structural model incorporates regression analysis to map the interrelationships between the latent variables and observable variables. This approach allows us to test our model hypotheses regarding how each variable affects ASDAS, either directly or indirectly. In general, in a methodologically sound SEM analysis, the theoretical model is defined before any statistical testing takes place. This model should only be modified if there is a sound theoretical justification. For this study, we hypothesized that there would be mainly direct effects of illness perception, central sensitization, physical activity and BMI on ASDAS, and indirect effects of psychological well-being, educational level and evasive coping strategies on ASDAS (Supplementary Fig. S1, available at *Rheumatology* online). Based on our previous study, we expected central sensitization to have the strongest effect, followed by illness perception [8].

Model fit was tested in R version 4.3.2 using the lavaan package version 0.6–17, and standardized coefficients were reported. The MLR estimator, a maximum likelihood estimator with robust (Huber-White) standard errors and a scaled test statistic was used. χ^2^/df (<5), comparative fit index (CFI) and Tucker Lewis Index (TLI) (≥0.90; ≥0.95), root mean square error of approximation (RMSEA) (≤0.08; ≤0.05) and standardized root mean square residual (SRMR) (<0.08) are indicative of acceptable and good model fit, respectively. Incremental modifications were considered to improve model fit inspired by modification indices. *P*-values of <0.05 were considered statistically significant. Prior to the SEM analysis, a confirmatory factor analysis (CFA) was performed to decide upon the inclusion of the ASDAS score itself *vs* a latent variable composed of ASDAS items. Finally, a power analysis was applied separately for identifying model fit and individually significant regression coefficients. Only complete cases were incorporated in the analysis.

### Ethics

This study complies with the Declaration of Helsinki. The GLAS cohort, as well as the amendment for this study, were approved by the ethics committees of the Medical Centre Leeuwarden and the University Medical Centre Groningen (TPO364/604). Written informed consent was obtained from all participating patients prior to enrolment into both GLAS and this study.

## Results 

From 2021 to 2023, 357 consecutive patients with axSpA were recruited, 25 were excluded due to incomplete data. Therefore, 332 patients were included in the analysis. Of these, 59% were male; median symptom duration was 21 years (interquartile range 11–32), mean ASDAS was 2.2 ± 0.9 and 54% used biological DMARDs (bDMARDs). See Table 1 for all baseline characteristics.

**Table 1.** Population characteristics

	Population (*n* = 332)
Age, years	49.8 ± 13.7
Female	135 (40.7%)
BMI, kg/m^2^	28.0 ± 5.95
Overweight (BMI 25–30 kg/m^2^)	111 (33.4%)
Obese (BMI >30 kg/m^2^)	101 (30.4%)
Smoking	78 (23.5%)
ISCED classification ≥5	117 (35.2%)
Symptom duration, years^a^	21 (11–32)
Diagnostic delay, years^b^	5 (1–12)
r-axSpA	228 (68.7%)
HLA-B27+	249 (75.2%)
Extraskeletal manifestations	136 (41.0%)
Psoriasis	35 (10.5%)
IBD	38 (11.4%)
Uveitis	90 (27.1%)
Current NSAID use	193 (58.1%)
Current bDMARD/tsDMARD use	178 (53.6%)
BASDAI	3.8 ± 2.1
≥4.0	148 (44.6%)
ASDAS	2.2 ± 0.86
≥2.1	168 (50.6%)
BASDAI Q2 (back pain; 0–10)	4 (2–7)
≥4	193 (58.1%)
BASDAI Q3 (peripheral pain/swelling; 0–10)	2 (0–5)
≥4	135 (40.7%)
BASDAI Q6 (back stiffness duration; 0–10)	2 (1–5)
≥4	122 (36.7%)
Patient GDA (0–10)	3 (2–6)
≥4	167 (50.3%)
CRP, mg/ml	2.0 (1.0–4.7)
≥5	82 (24.7%)
Physician GDA (0–10)	1 (1–2)
ASQoL (0–18)^c^	5 (1–9)
BASFI (0–10)^d^	3.0 (1.4–5.1)
Work disability >50% due to axSpA	51 (15.4%)
RDCI (0–9)	0 (0–2)
≥1	105 (31.6%)
Cardiovascular disease (including uncomplicated hypertension)	84 (25.3%)
mSQUASH	8935 (5145–11526)
CSI (0–100)	35.2 ± 14.5
PCS	13 (6–21)
IPQ-R	
Identity (0–14)	4 (3–6)
Consequences (6–30)	17.0 ± 5.44
Personal control (6–30)	20.4 ± 4.20
Treatment control (5–25)	18.0 ± 3.10
Illness coherence (5–25)	19 (16–23)
Emotional representations (6–30)	13 (10–16)
HADS	
Depression (0–21)	6 (3–9)
Score ≥11	48 (14.5%)
Anxiety (0–21)	4 (2–6)
Score ≥11	*22 (6.6%)*
CORS decreasing activity	19.8 ± 4.73

Values are *n* (%), mean ± s.d. or median (interquartile range). All missing values in study population <1% unless otherwise specified:

a 5.1%;

b 7.5%;

c 15.9%;

d 15.6%. ISCED: International Standard Classification of Education; r-axSpA: radiographic axial SpA; HLA-B27: Human Leucocyte Antigen B27; IBD: inflammatory bowel disease; bDMARD: biological DMARD; tsDMARD: targeted synthetic DMARD; GDA: global disease activity; CRP: C-reactive protein; ASQoL: Ankylosing Spondylitis Quality of Life questionnaire; RDCI: Rheumatic Disease Comorbidity Index; mSQUASH: modified Short QUestionnaire to ASsess Health-enhancing physical activity; CSI: Central Sensitization Inventory; PCS: Pain Catastrophizing Scale; IPQ-R: Revised Illness Perception Questionnaire; HADS: Hospital Anxiety and Depression Scale; CORS: COping with Rheumatic Stressors questionnaire.

### SEM analyses

#### Confirmatory factor analysis for ASDAS

We performed CFA for ASDAS on 332 axSpA patients from our standard-of-care cohort (Supplementary Fig. S2, Tables S1 and S2, available at *Rheumatology* online). The CFI was 0.994, TLI 0.988, RMSEA 0.044 (95% CI 0.000–0.108), SRMR 0.022 and χ^2^/df 1.6, indicating a good model fit. CRP did not have a significant factor loading, and therefore, ASDAS total score (instead of the separate components) was used in our SEM analysis.

#### Model modifications and fit

Model fit parameters of our hypothesized model (Supplementary Fig. S1, available at *Rheumatology* online) were unsatisfactory, and therefore the model was iteratively modified until arriving at the model shown in Fig. 1. During this iterative process, both timeline scales of the IPQ-R were removed from the measurement model because the scores lacked variation. Furthermore, the latent factor ‘coping’ was simplified to solely include the coping mechanism ‘reducing activities’, as the behavioural coping styles measured different constructs based on important differences in covariance with other variables. Regression paths were added from level of education to BMI, and from psychological well-being to CSI. Because of relatively large residual correlation between PCS, IPQ-R emotional representations and psychological well-being (HADS Anxiety and HADS Depression), the research team deliberated to make a modification while maintaining theoretical justification. Consequently, a new latent variable named ‘emotional amplification’ was introduced as a mediating variable between psychological well-being and illness perception. Together, the modifications described here improved model fit. For the final model, CFI was 0.936, TLI was 0.917, RMSEA was 0.057 (95% CI 0.045–0.070), SRMR was 0.065 and χ^2^/df was 2.0, indicating a satisfactory model fit.

#### Direct and indirect effects on ASDAS

ASDAS was significantly and positively associated with illness perception, CSI and BMI (Fig. 1, Tables 2 and 3). Perception had both a direct and large indirect positive effect via CSI on ASDAS. mSQUASH had an indirect negative effect via BMI. There was no significant indirect effect of mSQUASH through CSI on ASDAS. Psychological well-being had an indirect effect on ASDAS via emotional amplification and illness perception, and via CSI. Highest attained educational level had an indirect effect on ASDAS via illness perception and BMI.

**Table 2.** Structural equation modelling measurement model estimates

Factor loading	Estimate	Standardized estimate	95% CI	*P*
Psychological well-being				
HADS Anxiety	1.00	0.92		
HADS Depression	0.69	0.64	0.52–0.86	<0.001
Emotional amplification				
PCS	1.00	0.82		
Emotional representations	0.94	0.76	0.79 to 1.09	<0.001
Illness perception				
Identity	1.00	0.70		
Consequences	0.92	0.64	0.72–1.13	<0.001
Illness coherence	–0.56	–0.39	–0.76 to –0.36	<0.001
Personal control	–0.46	–0.32	–0.63 to –0.29	<0.001
Treatment control	–0.53	–0.36	–0.70 to –0.35	<0.001

HADS: Hospital Anxiety and Depression Scale; PCS: Pain Catastrophizing Scale.

**Table 3.** Structural equation modelling structural model estimates; R^2^ equals 1 minus standardized residual variance

Regression	Estimate (B)	Standardized estimate (β)	95% CI	*P*
Emotional amplification	R^2^: 0.41			
Psychological well-being	0.57	0.64	0.37–0.76	<0.001
Illness perception	R^2^: 0.55			
Level of education	–0.34	–0.24	–0.50 to –0.19	<0.001
Emotional amplification	0.60	0.70	0.482–0.72	<0.001
Decreasing activity	R^2^: 0.22			
Illness perception	0.68	0.47	0.51–0.86	<0.001
mSQUASH	R^2^: 0.12			
Decreasing activity	–0.35	–0.34	–0.45 to –0.24	<0.001
CSI	R^2^: 0.69			
Illness perception	1.01	0.71	0.84–1.18	<0.001
mSQUASH	–0.07	–0.07	–0.14 to 0.01	0.079
Psychological wellbeing	0.21	0.20	0.08–0.34	0.001
BMI	R^2^: 0.07			
mSQUASH	–0.19	–0.19	–0.28 to –0.09	<0.001
Level of education	–0.39	–0.19	–0.59 to –0.18	<0.001
ASDAS	R^2^: 0.36			
Illness perception	0.50	0.35	0.16–0.85	0.004
CSI	0.24	0.24	0.01–0.47	0.040
BMI	0.19	0.19	0.10–0.28	<0.001
mSQUASH	0.05	0.05	–0.04 to 0.13	0.273

mSQUASH: modified Short QUestionnaire to ASsess Health-enhancing physical activity; CSI: Central Sensitization Inventory; ASDAS: Axial Spondyloarthritis Disease Activity Score.

The graphical representations of the final measurement model and structural model are shown in Fig. 1A and B, respectively. The residual variances of the model and its modification indices are shown in Supplementary Tables S3 and S4, available at *Rheumatology* online, respectively.

#### Statistical power

*A posteriori* power calculation showed a power of >99% for identifying a model with RMSEA <0.8. Power for identifying individually significant regression coefficients ranged from 0.40–1.00 (Supplementary Table S5, available at *Rheumatology* online). Sensitivity analysis after exclusion of six multivariate outliers (based on robust Mahalanobis distance, Stahel-Donoho estimator) yielded similar fit parameters and no differences in statistical significance of all results. All analyses were replicated in Mplus, which yielded almost identical results to those described above, demonstrating the robustness of the analyses.

## Discussion

In this cross-sectional study within a standard-of-care cohort of treated axSpA patients with long-term symptom duration, a biopsychosocial model of the interrelationships of ASDAS with central sensitization, psychosocial and lifestyle factors was tested using SEM to better understand and interpret the multifaceted nature of assessing DA with ASDAS in axSpA. Illness perception showed the strongest direct association, while central sensitization, BMI and physical activity also showed significant direct associations with ASDAS.

Illness perceptions appear to be influenced by patients’ psychological well-being and emotional factors, independently of educational level, as shown by the high, significant regression coefficients (sequentially β = 0.64 and β = 0.70; see Fig. 1). Due to the strong associations of illness perception with psychological well-being and emotional factors, illness perceptions cannot be fully understood without considering emotional and psychological well-being in axSpA [32]. central sensitization was very strongly influenced by illness perception (β = 0.71), which aligns with the current understanding of chronic pain [33]. In this model, illness perception can be interpreted as an important factor of the descending pain-modulatory mechanisms involved in central sensitization [34]. Our results support the hypothesis that unfavourable ideas about their disease invoke a non-conscious tendency towards increased central sensitivity in patients with axSpA.

Central sensitization also had a direct effect on ASDAS, independent of other variables (β = 0.24). This is consistent with previous studies showing that axSpA patients with signs of central sensitization have higher ASDAS scores [8, 35]. Central sensitization causes increased regional or widespread pain, other types of sensory hypersensitivity and fatigue [36], all mediating the influence of central sensitization on ASDAS. While the CSI is not a direct assessment of central sensitization, it can be used for screening purposes and serves as an appropriate proxy for central sensitization. With the cutoff score of ≥40, the CSI has 81% sensitivity and 75% specificity for syndromes strongly associated with central sensitization such as chronic low back pain, FM and irritable bowel syndrome [37]. The recently published consensus recommendations [38] and clinical criteria for nociplastic pain [36] may provide better guidance in differentiating nociplastic pain from nociceptive pain and neuropathic pain. However, more empirical research is needed to validate these recommendations in axSpA.

In the final SEM model, ‘emotional amplification’, composed of pain-related worrying (PCS) and negative emotions towards their disease (IPQ-R emotional representations) had an indirect effect on ASDAS. An association between negative emotions and ASDAS has previously been demonstrated [8, 39]. Interestingly, in RA, pain-related worrying was only associated with patient reported outcomes and not with objective markers of inflammation (swollen joint count, CRP level and abnormalities on US) [40]. So far, this has not yet been investigated directly in axSpA. A study in RA reported that pain-related worrying may interact with psychological well-being indirectly influencing pain perception [41]. Moreover, in axSpA, depression specifically was found to be associated with higher ASDAS [42]. In the present study, we showed that psychological well-being indirectly affects illness perception by influencing pain- and disease-related emotions. Furthermore, psychological well-being also affected central sensitization directly. This is unsurprising, considering that a diagnosis of depression is included in the second part of the CSI, which inquires after the diagnosis of central sensitization–associated syndromes [22].

Highest attained educational level showed a significant direct association with illness perception (β = –0.24), indicating that more educated patients had more positive illness perceptions resulting in a lower ASDAS. There was also a significant direct association between level of education and BMI (β = –0.19) suggesting that a healthier weight is another pathway through which level of education affects ASDAS. Patients with lower educational levels have on average a lower socioeconomic status, which can lead to limited access to healthy lifestyle such as healthy foods. They also have overall lower health literacy and food literacy [43].

Concerning lifestyle factors, physical activity had no direct effect on ASDAS, meaning that, after correction for the other variables in the model, more physical activity does not lead to a lower ASDAS. This was an unexpected finding, considering it is well established that exercise improves disease outcomes, including inflammation [16] and DA assessments [44]. This has led to exercise being a cornerstone in the management of axSpA [4]. However, there was an uncorrected correlation between physical activity and ASDAS of –0.17, showing that more physically active patients have a lower ASDAS. This possibly indicates that the beneficial effect of physical activity on ASDAS is mediated by other variables in the model (e.g. BMI, or illness perception and central sensitization). Physical activity may have an indirect beneficial effect on ASDAS through reinforcement of beneficial illness perceptions [13]. On the other hand, activity patterns and sedentary patterns [45], including the type of physical activity (for example, blue-collar *vs* white-collar jobs [46]) may be more important than total weekly physical activity levels. However, we found a lower correlation between physical activity and ASDAS (–0.17) than in other studies using the mSQUASH (–0.27) [31]. This might be caused by ‘questionnaire fatigue’, considering mSQUASH was the last instrument completed by the study participants in a long series of questionnaires. Expanding on lifestyle, we confirmed previous findings that overweight and obesity are very prevalent in axSpA [47], and that they are related to ASDAS [8, 48]. (Lifestyle) interventions aimed at weight reduction can alleviate symptoms and inflammatory burden and should therefore be a point of concern in overweight axSpA patients.

This study provides a biopsychosocial framework that helps to understand and identify variables related to high ASDAS irrespective of anti-inflammatory treatment, which should be addressed in the further management of axSpA. Illness perception had the largest potential effects on ASDAS, suggesting that overall, the greatest benefit in daily clinical practice in patients already treated for axSpA and an ASDAS reflecting high disease activity may result from addressing negative illness perception and improving psychological well-being. Patient education is an important tool for targeting illness perception. Effective patient education requires a patient-centred approach, in which illness perceptions have to be taken into account [49]. Therefore, upon diagnosis and during follow-up, evaluation of patients’ illness perceptions seems appropriate. The effect on ASDAS of illness perception is closely followed by the effect on ASDAS of central sensitization. Potential treatments targeting both illness perceptions and central sensitization, proven to be effective in randomized controlled trials in patients with chronic spinal pain, are cognitive functional therapy [50], graded sensorimotor retraining [51] and pain neuroscience education combined with cognition-targeted motor control training [13]. These may also be possible treatment options in axSpA. At the same time, evaluation for psychological comorbidities may also be indicated. In our study, 14.5% of participants had a probable depression and 6.6% had a probable anxiety disorder according to the HADS. Both can significantly affect ASDAS and also treatment outcome [52]. Altogether, our data in combination with the results of previous studies underscore the need for a broad holistic biopsychosocial approach in the management of patients with axSpA.

This is the first large study conducting SEM, building a biopsychosocial model of variables related to DA (ASDAS) in a standard-of-care cohort of axSpA patients. In this cohort of treated patients with long-term disease, the overall inflammation related to axSpA can be assumed to be low, due to overall low CRP, high percentage of patients on targeted synthetic DMARDs and bDMARDs, low numbers of swollen joints, and low physician-assessed global disease activity (GDA), while patient GDA was overall experienced as moderate. Patients rate DA mostly on (subjective) symptoms while physicians also take objective measures into account [53]. Additionally, in the CFA of ASDAS a very good model fit was found (demonstrating good structural validity) with a factor loading of 0 for CRP. Furthermore, our presented model is grounded on a large body of combined literature for axSpA and other (inflammatory rheumatic) diseases and syndromes. Furthermore, SEM allowed for a unique multidimensional approach to test our hypotheses.

It should be noted that almost all patients included have a long symptom duration and are treated according to the current standard-of-care, which means that our results are not representative of patients with more recently diagnosed axSpA and untreated inflammatory disease. Due to the methodological design of SEM in combination with the statistical power requirements, we were unable to include variables that could have potentially improved our model. For example, sex could probably best be studied by building two separate models for male and female patients in order to account for expected important effect modifications within the original model. Furthermore, different comorbidities could influence the interrelationships of more than one of the studied variables. Including comorbidities in the model would therefore probably result in an increased complexity of the model. Since the number of comorbidities in our cohort was low, the influence on the model as a whole is expected to be small. Therefore, we did not add comorbidities into our model. In addition, radiographic axSpA and spinal outcome may have accounted for some of the symptoms also included in ASDAS, and therefore may have unintentionally influenced our results. We know from the scientific literature that degenerative changes shown on imaging of the spine do not correlate well with experienced symptoms such as pain [54]. Furthermore, disease burden of non-radiographic axSpA and radiographic axSpA are similar. So, in general, we do not expect this to have a large influence on our results.

Finally, sleep quality and health literacy may also improve the multidimensional understanding of DA measured with ASDAS. Nevertheless, even without these variables, our study still provides very relevant insights into the interpretation of ASDAS in daily clinical practice.

## Conclusion

In daily clinical practice, we are faced with the reality that only approximately half of axSpA patients treated with anti-inflammatory therapy such as bDMARDs achieve a low disease activity state or remission according to ASDAS. Since ASDAS is an algorithm based on patient-reported outcomes and CRP, it is clinically relevant to have insight into the factors influencing ASDAS in this situation. Based on this situation we have built a theoretical model which was analysed using SEM. This SEM analysis conducted in our standard-of-care cohort of patients with axSpA from daily clinical practice has provided a better understanding of the interrelationships between ASDAS, central sensitization, and psychosocial and lifestyle factors. These results showed that ASDAS does not only reflect disease-related inflammation. Psychosocial and lifestyle factors, in particular illness perception and central sensitization, should be taken into account when interpreting ASDAS. These results will help researchers and clinicians to better understand how psychosocial and lifestyle factors are related to ASDAS and will guide clinicians and patients towards a more biopsychosocial approach to the management of axSpA.

## Supplementary Material

keaf102_Supplementary_Data

## Data Availability

The data underlying this article will be shared on reasonable request to the corresponding author. Concise R code for the primary and sensitivity analyses is available online at: https://github.com/sckieskamp/SEM-analysis-R-code/blob/7e5b42f2205e73269c9f710d433626b6756aa677/R%20code.
