# Supplementary material for: Interrelationships of disease activity, central sensitization, psychosocial and lifestyle factors in axial spondyloarthritis
Source: Rheumatology (Oxford). 2025 Feb 22;64(6):3547–55. doi: 10.1093/rheumatology/keaf102 (PMC12107071; doi:10.1093/rheumatology/keaf102)
Supplement: keaf102_Supplementary_Data [file keaf102_supplementary_data.docx]

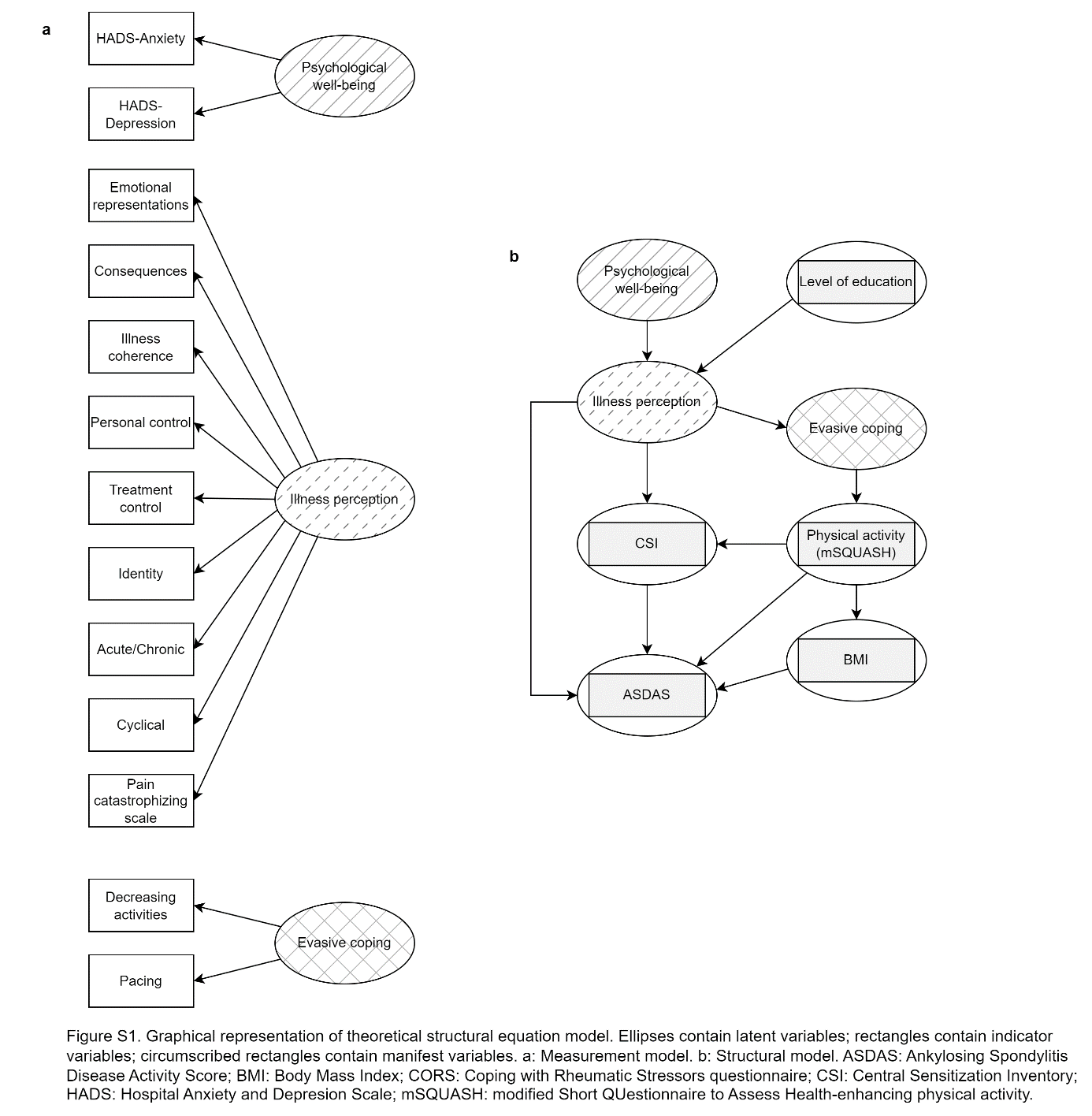


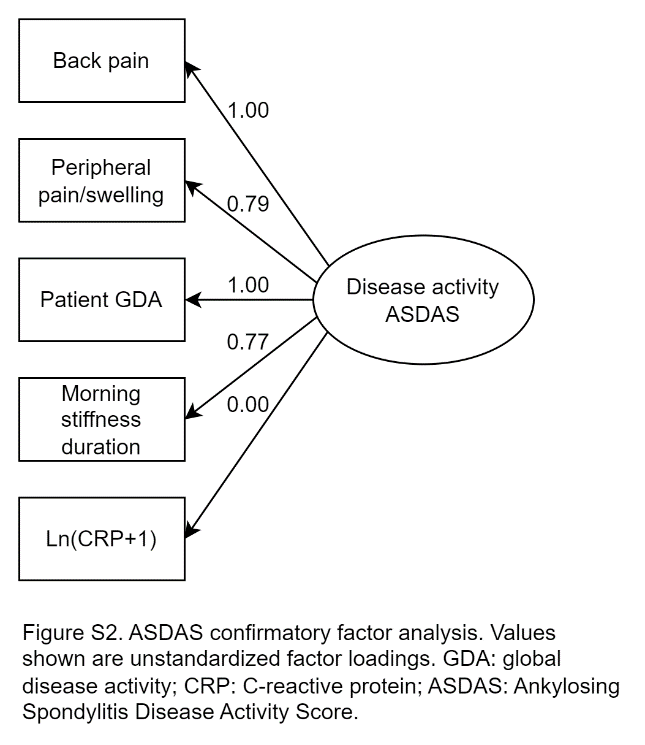


|  | Original ASDAS factor weight ratio | CFA estimate | CFA estimate, standardized | 95% CI for CFA estimate | p |
| --- | --- | --- | --- | --- | --- |
| Patient global | 1.00 | 1.00 | 0.88 | N/A | N/A |
| Back pain | 1.09 | 1.00 | 0.88 | 0.89 – 1.12 | <0.001 |
| Peripheral pain/swelling | 0.64 | 0.79 | 0.63 | 0.66 – 0.92 | <0.001 |
| Morning stiffness duration | 0.55 | 0.77 | 0.60 | 0.63 – 0.90 | <0.001 |
| Ln(CRP+1) | 5.27 | 0.00 | 0.01 | -0.03 – 0.03 | 0.823 |
| Table S1. ASDAS confirmatory factor analysis (CFA) factor loadings. CRP: C-reactive protein. | | | | | |

|  | Residual variance | Residual variance, standardized |
| --- | --- | --- |
| Patient global | 1.48 | 0.23 |
| Back pain | 1.47 | 0.23 |
| Peripheral pain/swelling | 4.61 | 0.61 |
| Morning stiffness duration | 4.96 | 0.64 |
| Ln(CRP+1) | 0.30 | 1.00 |
| ASDAS | 4.82 | N/A |
| Table S2. Residual variances in ASDAS confirmatory factor analysis. Standardized residual variance is equal to 1 minus R^2^. CRP: C-reactive protein; ASDAS: Axial Spondyloarthritis Disease Activity Score. | | |

| Variable | Residual variance | Residual variance, standardized (1-R^2^) |
| --- | --- | --- |
| Psychological well-being (lv) | N/A | N/A* |
| Perception (lv) | 0.18 | 0.39 |
| Emotional amplification (lv) | 0.36 | 0.55 |
| Level of education | N/A | N/A* |
| CSI | 0.29 | 0.30 |
| CORS Decreasing activities | 0.78 | 0.79 |
| mSQUASH | 0.92 | 0.92 |
| BMI | 0.93 | 0.94 |
| ASDAS | 0.63 | 0.64 |
|  |  |  |
| HADS Anxiety | 0.18 | 0.18 |
| HADS Depression | 0.58 | 0.58 |
| IPQ-R Identity | 0.51 | 0.53 |
| IPQ-R Consequences | 0.58 | 0.59 |
| IPQ-R Illness coherence | 0.84 | 0.84 |
| IPQ-R Personal control | 0.66 | 0.67 |
| IPQ-R Treatment control | 0.52 | 0.53 |
| IPQ-R Emotional representations | 0.42 | 0.42 |
| PCS | 0.35 | 0.35 |
| Table S3. Residual variances in SEM model. *Exogenous variable. CSI: Central Sensitization Inventory; CORS: COping with Rheumatic Stressors questionnaire; mSQUASH: modified Short QUestionnaire to ASsess Health-enhancing physical activity; BMI: body mass index; ASDAS: Axial Spondyloarthritis Disease Activity Score; HADS: Hospital Anxiety and Depression Scale; IPQ-R: Revised Illness Perception Questionnaire; PCS: Pain Catastrophizing Scale. | | |

| Suggested alteration | Modification index | Expected parameter change (EPC) | Standardized EPC |
| --- | --- | --- | --- |
| Emotional amplification =~ Illness coherence | 21.54 | -0.54 | -0.44 |
| Illness identity ~~ Central sensitization | 19.10 | 0.16 | 0.42 |
| Emotional representations ~~ Illness coherence | 15.18 | -0.15 | -0.25 |
| Emotional amplification ~ Level of education | 14.64 | -0.35 | -0.43 |
| Perception ~ Physical activity | 11.63 | -0.15 | -0.22 |
| Perception =~ HADS anxiety | 10.27 | -0.38 | -0.26 |
| Table S4. All modification indices >10 for SEM model. Operators: =~ (factor loading), ~ (regression), ~~ (correlated residuals). HADS: Hospital Anxiety and Depression Scale. | | | |

| Regression | Simulated power |
| --- | --- |
| 🡪Emotional amplification |  |
| Psychological well-being | 1.00 |
| 🡪Illness perception |  |
| Level of education | 0.99 |
| Psychological well-being | 1.00 |
| 🡪Decreasing activity |  |
| Illness perception | 1.00 |
| 🡪mSQUASH |  |
| Decreasing activity | 1.00 |
| 🡪CSI |  |
| Illness perception | 1.00 |
| mSQUASH | 0.48 |
| Psychological well-being | 0.97 |
| 🡪BMI |  |
| mSQUASH | 0.94 |
| Level of education | 0.95 |
| 🡪ASDAS |  |
| Illness perception | 0.90 |
| CSI | 0.64 |
| BMI | 0.99 |
| mSQUASH | 0.19 |
| Table S5. Post-hoc power analysis for calculated parameter estimates. Sample size 332, alpha level 0.05, simulation seed 42, 2000 simulations. mSQUASH: modified Short QUestionnaire to ASsess Health-enhancing physical activity; CSI: Central Sensitization Inventory; BMI: body mass index; ASDAS: Axial Spondyloarthritis Disease Activity Score. Power calculation: Wang YA, Rhemtulla M. Power analysis for parameter estimation in structural equation modeling: A discussion and tutorial. Adv. Meth. Pract. Psychol. Sci. 2021 Jan;4(1). | |
